# Correlation of Non-Invasive Transthoracic Doppler Echocardiography with Invasive Doppler Wire-Derived Coronary Flow Reserve and Their Impact on Infarct Size in Patients with ST-Segment Elevation Myocardial Infarction Treated with Primary Percutaneous Coronary Intervention

**DOI:** 10.3390/jcm13092484

**Published:** 2024-04-24

**Authors:** Dejan Milasinovic, Milorad Tesic, Olga Nedeljkovic Arsenovic, Ruzica Maksimovic, Dragana Sobic Saranovic, Dario Jelic, Milorad Zivkovic, Vladimir Dedovic, Stefan Juricic, Zlatko Mehmedbegovic, Olga Petrovic, Danijela Trifunovic Zamaklar, Ana Djordjevic Dikic, Vojislav Giga, Nikola Boskovic, Marija Klaric, Stefan Zaharijev, Lazar Travica, Djordje Dukic, Djordje Mladenovic, Milika Asanin, Goran Stankovic

**Affiliations:** 1 Department of Cardiology, University Clinical Center of Serbia, 11000 Belgrade, Serbia; milasin.d18@gmail.com (D.M.); duga13@gmail.com (D.J.); mzivkovic05@hotmail.com (M.Z.); vladeda@gmail.com (V.D.); stefan.juricic@gmail.com (S.J.); zlatkombegovic@gmail.com (Z.M.); danijelatrif@gmail.com (D.T.Z.); voja2011@yahoo.com (V.G.); belkan87@gmail.com (N.B.); marija.klaric95@gmail.com (M.K.); zaharijev@gmail.com (S.Z.); lazartravica18@gmail.com (L.T.); djoleczv1994@gmail.com (D.D.); mladendjolem@gmail.com (D.M.); masanin2013@gmail.com (M.A.); 2 Faculty of Medicine, University of Belgrade, 11000 Belgrade, Serbia; olganedeljkovic@gmail.com (O.N.A.); rmaksimovic@yahoo.com (R.M.); dsobic2@gmail.com (D.S.S.); 3 Center for Radiology and Magnetic Resonance, University Clinical Center of Serbia, 11000 Belgrade, Serbia; 4 Center for Nuclear Medicine with PET, University Clinical Center of Serbia, 11000 Belgrade, Serbia

**Keywords:** coronary microcirculation, coronary flow reserve, STEMI, primary PCI, infarct size

## Abstract

**Background**: Coronary microvascular dysfunction is associated with adverse prognosis after ST-segment elevation myocardial infarction (STEMI). We aimed to compare the invasive, Doppler wire-based coronary flow reserve (CFR) with the non-invasive transthoracic Doppler echocardiography (TTDE)-derived CFR, and their ability to predict infarct size. **Methods**: We included 36 patients with invasive Doppler wire assessment on days 3–7 after STEMI treated with primary percutaneous coronary intervention (PCI), of which TTDE-derived CFR was measured in 47 vessels (29 patients) within 6 h of the invasive Doppler. Infarct size was assessed by cardiac magnetic resonance at a median of 8 months. **Results**: The correlation between invasive and non-invasive CFR was modest in the overall cohort (rho 0.400, *p* = 0.005). It improved when only measurements in the LAD artery were considered (rho 0.554, *p* = 0.002), with no significant correlation in the RCA artery (rho −0.190, *p* = 0.435). Both invasive (AUC 0.888) and non-invasive (AUC 0.868) CFR, measured in the recanalized culprit artery, showed a good ability to predict infarct sizes ≥18% of the left ventricular mass, with the optimal cut off values of 1.85 and 1.80, respectively. **Conclusions**: In patients with STEMI, TTDE- and Doppler wire-derived CFR exhibit significant correlation, when measured in the LAD artery, and both have a similarly strong association with the final infarct size.

## 1. Introduction

Coronary flow reserve (CFR) is the ratio between maximal and resting blood flow and it shows the ability of coronary circulation to increase flow to the myocardium under conditions of stress or when vasodilating agents are administered [1]. Changes in CFR may reflect disease processes that affect both epicardial and microvascular compartments of the coronary tree [1,2]. Impaired CFR has recently been associated with increased incidence of both mortality and non-fatal cardiovascular events in a range of diseases affecting the heart, from manifest coronary artery disease (CAD), including reperfused ST-elevation myocardial infarction (STEMI) and ischemia with non-obstructive coronary arteries (INOCA), to heart failure and diabetes mellitus without symptoms of CAD [3]. In patients with STEMI in whom early revascularization is recommended [4], low CFR values in infarct-related arteries after recanalization have been associated with adverse remodeling of the left ventricle [5]. Moreover, it was previously recognized that the information provided by measuring CFR in the recanalized vessel offers more prognostic capacity in terms of left ventricular recovery compared with angiographic surrogates such as TIMI flow grade/count and myocardial blush grade [6]. Measuring CFR in the infarct-related artery, shortly after vessel opening, showed that CFR values ≤ 1.3 were associated with a higher risk of adverse cardiac events over 5 years after STEMI [7]. Specifically, low CFR values were linked to lower left ventricular ejection fractions and a higher incidence of heart failure [6]. Importantly, when assessed at 4–8 days after recanalization of the occluded culprit artery in the setting of STEMI, CFR was associated with the extent of microvascular injury on cardiac magnetic resonance (CMR) [8]. Patients with severe microvascular obstruction had mean CFR values of 1.6, whereas those with no microvascular obstruction on CMR had a mean CFR of 2.0 [8]. Of note, measuring CFR in the patent non-culprit artery of STEMI patients has also provided prognostic information, with values ≤2.0 being associated with a four-fold higher risk of cardiac mortality during 10-year follow-up [9].

In most of the hitherto published studies documenting the predictive value of CFR in the culprit and non-culprit arteries after STEMI, invasive methods to measure or estimate coronary flow velocity at baseline and during pharmacologically induced hyperemia, mostly with adenosine or papaverine, were applied. Peak coronary flow velocity can be directly measured with a Doppler-tipped coronary guidewire, which produces Doppler-derived CFR [10]. On the other hand, thermodilution-derived CFR relies on the estimation of blood flow by calculating the mean transit time of intracoronary bolus injections of saline with a dedicated coronary guidewire equipped with a thermistor that measures temperature difference as the saline passes from the proximal to distal part of the coronary tree [11]. Recently, a continuous thermodilution method was described using the same thermistor-equipped coronary wire and continuous saline infusion through a dedicated microcatheter [12]. Unlike with Doppler wires and thermodilution, where pharmacological induction of hyperemia is needed to obtain CFR, continuous thermodilution assesses absolute flow in mL/min by means of the continuous intracoronary saline infusion. Apart from the described invasive methods, coronary flow can also be measured non-invasively by positron emission tomography (PET), CMR, and transthoracic Doppler echocardiography (TTDE) [1]. While the use of CMR to quantify myocardial blood flow (MBF) is still in its infancy, PET is considered a gold-standard method to non-invasively assess CFR by quantifying MBF at rest and during pharmacologically induced hyperemia [1,13]. Impaired CFR (<2) in PET studies has been associated with higher cardiac mortality risk [14]. The most widely available method to non-invasively assess CFR is transthoracic Doppler echocardiography (TTDE), which measures peak diastolic flow velocity at rest and during hyperemia [15], similar in principle to the above-described invasive Doppler wire-based technology. TTDE measured in the LAD territory was shown to agree with both global and LAD-specific myocardial blood flow reserve on PET [16]. In terms of its prognostic value, impaired TTDE-derived CFR was associated with the presence of significant CAD on angiography and was predictive of MACE and mortality in chronic coronary syndromes [17,18,19]. Although several small, previous studies did demonstrate the ability of TTDE-assessed coronary flow velocity and its derived parameters, such as diastolic deceleration time and systolic coronary flow reversal, to predict adverse LV remodeling or perfusion defects on SPECT after STEMI [20,21,22], most contemporary studies which associate impaired CFR and other indices of coronary microvascular dysfunction with CMR-assessed infarct size and clinical outcomes utilized invasive methods, either with Doppler wires or thermodilution [23,24].

Given the widespread availability of transthoracic Doppler echocardiography, this non-invasive method to measure CFR may provide an important clinical tool to stratify patients after primary PCI according to their risk of heart failure and mortality. However, the evidence confirming the correlation of non-invasive TTDE CFR with invasively derived CFR in patients with STEMI treated by primary PCI is lacking. Moreover, a recent study showed no correlation between non-invasive TTDE CFR and invasive thermodilution-derived CFR in post-PCI patients [25].

Our study therefore aims to explore the correlation between non-invasive TTDE-derived and invasive Doppler wire-derived coronary flow reserve in patients with STEMI and their respective association with CMR-assessed infarct size and LV function.

## 2. Materials and Methods

### 2.1. Patient Population and Study Design

This was a prospective study enrolling patients with acute ST-segment elevation myocardial infarction who underwent an angiographically successful primary PCI within 12 h of the onset of symptoms, between January 2020 and February 2024. Inclusion criteria were (1) diagnosis of STEMI, based on chest pain lasting >30 min and ST-segment elevation in ≥2 contiguous leads; (2) the presence of TIMI 0 or TIMI 1 coronary flow in the infarct-related artery on initial angiography; and (3) a successful primary PCI, defined as TIMI 3 flow with residual stenosis < 30% after stenting. Exclusion criteria were (1) previous history of CAD or heart failure, (2) previous PCI or CABG, (3) hemodynamic instability, (4) malignant arrhythmia, (5) Killip class ≥ 2 on admission to hospital, (6) critical stenosis of a non-infarct-related artery on initial angiography, (7) an inability to receive DAPT for 12 months, and (8) an inability to undergo CMR.

All patients were treated with loading doses of aspirin (300 mg) and a P2Y12 inhibitor (either 180 mg of Ticagrelor or 600 mg of clopidogrel) prior to primary PCI and intravenous heparin during the procedure. Transradial access utilizing 6 French guide catheters was the default approach. All patients were treated with an implantation of a drug eluting stent in the culprit lesion and a 12-month dual antiplatelet therapy. The study protocol was approved by the local ethics committee and all patients provided written informed consent for their participation in this study.

### 2.2. Invasive Measurements of Coronary Physiology Indices with Doppler Wire

An invasive assessment of coronary physiology indices in the infarct-related artery was performed immediately following successful stent implantation in the culprit lesion. A repeated invasive assessment, which is the subject of this manuscript, was performed on days 3–7 after primary PCI in both the IRA and a non-infarct-related reference artery when possible. For this purpose, a dual sensor guidewire (ComboWire, Philips Volcano, San Diego, CA, USA), which simultaneously records distal coronary artery pressure (Pd) and Doppler flow velocity, was used. In addition, electrocardiographic recording and aortic pressure were simultaneously obtained by the ComboMap system (Philips Volcano, San Diego, CA, USA). Aortic pressure (Pa) was measured at the tip of the guiding catheter. After equalizing with the aortic pressure, the Doppler wire was positioned distal to the stented coronary artery segment, and the best Doppler signal was searched for by rotating the wire mid lumen. CFR was then calculated as a ratio of directly measured flow velocity during hyperemia and at rest. In addition, fractional flow reserve (FFR) and hyperemic microvascular resistance (HMR) were calculated from the simultaneous pressure and flow velocity measurements. HMR was the ratio between distal coronary pressure and flow velocity and FFR was the ratio of distal coronary pressure to aortic pressure, both during hyperemia. Hyperemia was achieved by intracoronary injection of at least 150 mcg of adenosine.

### 2.3. Coronary Flow Velocity Measurement with Transthoracic Doppler Echocardiography

Transthoracic Doppler echocardiography to determine coronary flow velocity reserve was performed on the same day and within 6 h of the invasive intracoronary Dop-pler wire assessment on days 3–7 after primary PCI. The investigators who performed the TTDE were blinded to the results of the invasive, intracoronary Doppler wire measurements, and the other way around if TTDE preceded invasive CFR assessment. A digital ultrasound system (Acuson SC2000 Prime; Siemens Medical Solutions USA, Mountain View, CA, USA) and the GE Healthcare Vivid E9 ultrasound system (GE Vingmed Ultrasound AS, Horten, Norway) with multifrequency probes were used. Both the distal segment of the left anterior descending (LAD) artery and the posterior descending branch of the right coronary artery (RCA) were evaluated with a 4 MHz transducer. The velocity range was set at 16–24 cm/s. The distal segment of the LAD artery was looked for in a modified three-chamber view, whereas the flow velocity in the posterior descending branch of the RCA artery was assessed in the apical two-chamber view, by slightly rotating anticlockwise and tilting the probe anteriorly, until the coronary blood flow became identifiable in the posterior interventricular groove. A pulsed wave with a sample volume width of 3 to 5 mm was used to measure coronary flow velocity. The ultrasound beam was aligned in parallel with the coronary flow maintaining a stable transducer position at rest and during hyperemia, which was achieved by adenosine infusion (0.14 mg/kg/min intravenously, during 2 min). We averaged the results from 3 coronary flow measurements both at rest and during hyperemia. CFR was then expressed as the ratio between hyperemic and rest peak diastolic flow velocities. Our center previously reported an interobserver agreement of 96% [26].

### 2.4. Cardiac Magnetic Resonance Imaging

As per protocol, CMR was performed at 3–12 months after primary PCI using a 1.5 Tesla MR scanner (Magnetom Avanto, Siemens, Erlangen, Germany) with a phased-array coil. ECG-gated cine imaging was used with breath-holding in 3 standard long-axis chamber vies (2, 3, and 4). Short-axis images were acquired utilizing ECG-gated steady-state free precession (SSFP) cine images for functional analysis. Short-axis cine images were ac-quired as a stack from the mitral valve plane through the apex covering the entire ventricles; the slice thickness was 8 mm, and the field of view was 360 mm × 306 mm. Late gadolinium enhancement (LGE) images were obtained 10–15 min after administration of 0.2 mmol/kg of Gadobutrol (Gadovist, Bayer Inc., Mississauga, ON, Canada) using phase-sensitive inversion recovery sequence (PSIR). PSIR was conducted in SAX view covering the whole ventricle with a slice thickness of 6 mm, and also in 2-, 3-, and 4-chamber views. To obtain LGE images, a reference region in the remote myocardium was marked, and a signal intensity above 2 standard deviations of the mean intensity of this remote region was used to quantify LGE. The CMR readers (RM, ONA) were blinded to the results of the intracoronary and echocardiographic measurements. Instances of disagreement when performing CMR measurements were resolved by consensus. The analyses provided left ventricular ejection fractions and end-diastolic and end-systolic myocardial volumes. In short-axis LGE images, the total infarct size was obtained by dividing the infarct mass by the total left ventricular mass.

### 2.5. Statistics

Continuous variables with normal distribution according to the Shapiro–Wilk test were described as means (±standard deviation) and were compared using the Student *t*-test. For variables with non-normal distribution, medians (± interquartile range) were used and the Mann–Whitney U test for comparisons. Categorical variables were presented as counts and compared with the Chi squared test (or the Fischer exact test if expected cell counts were <5). To assess correlations between continuous variables, we used the Pearson (r) or, when needed, Spearman (rho) correlation coefficient. The concordance between TTDE CFR and invasive Doppler wire-derived CFR was evaluated by Bland–Altman plot analysis. Receiver-operating characteristic (ROC) curves were constructed to assess the capacity of non-invasive TTDE and invasive Doppler wire-derived CFR to predict the final infarct size ≥18% of the LV and left ventricular ejection fraction <40%. The ROC curves were compared with the Delong method, and the Youden index was used to identify optimal cut off values. For all comparisons, *p*-values < 0.05 were considered significant. The sample size for our study was based on previous similar comparisons between invasive and non-invasive CFR, which included 25 and 23 patients [27,28]. A formal size calculation was performed for a prediction model that utilized the ROC curve to test the ability of CFR to predict large infarct sizes. Given a two-sided alpha of 0.05, 24 patients would be needed to have a power of 80% to detect an area under the curve (AUC) of 0.85, with the null hypothesis value of AUC of 0.5, and defining large infarct size as ≥18% of the LV. All statistical analyses were performed with SPSS 22.0 (IBM, Chicago, IL, USA).

## 3. Results

### 3.1. Patient Cohort and Baseline Characteristics

A total of 36 patients who had analyzable invasive intracoronary physiology measurements, performed at a median of 4 days (3.0–6.0) after primary PCI, were included. Invasive Doppler wire-derived CFR was compared with non-invasive TTDE-derived CFR in 47 vessels (29 patients). Non-invasive CFR could not be obtained in three vessels (two RCA and one LAD) from three different patients due to poor TTDE signals. Overall, seven patients with invasive coronary physiology measurements did not undergo TTDE due to logistical constraints during the COVID-19 pandemic. CMR-based final infarct size quantification was available for 31 patients at a median of 8 months (6.0–8.0) following the acute event. Baseline clinical characteristics and the presentation of the included patients are presented in Table 1.

### 3.2. Correlation between Invasive and Non-Invasive CFR

**Figure 1:**
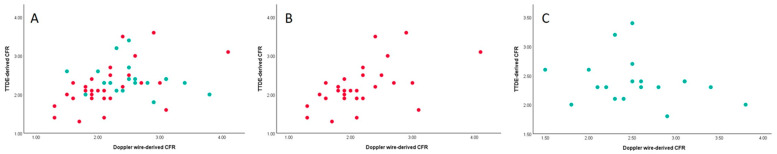
Correlation between invasive, Doppler wire-derived and non-invasive, transthoracic Doppler echocardiography-derived coronary flow reserve in patients with ST-segment elevation acute myocardial infarction. (**A**) Overall population including measurements in both LAD (red dots) and RCA (green dots) arteries. (**B**) Significant correlation between invasive and non-invasive coronary flow reserve in the LAD artery. (**C**) Lack of significant correlation between invasive and non-invasive coronary flow reserve in the RCA artery.

**Figure 2:**
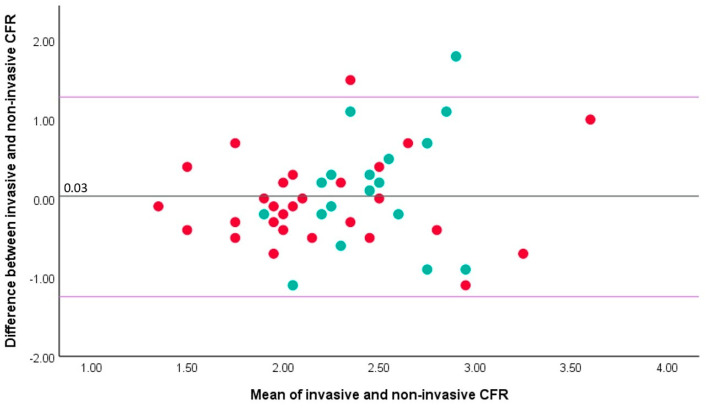
Bland–Altman graph showing similar pattern of agreement over a range of CFR values in the LAD (red dots) and RCA (green dots) arteries.

There was significant but moderate correlation between non-invasive TTDE-derived CFR and invasive Doppler-derived CFR in the overall cohort (rho 0.40, *p* = 0.005) (Figure 1A). Of the 47 vessels tested, 28 were LAD and 19 were RCA. Whilst non-invasive TTDE-derived CFR in the LAD artery did significantly correlate with invasive CFR (rho 0.55, *p* = 0.002; Figure 1B), there was no such correlation for the RCA artery (rho −0.19, *p* = 0.44; Figure 1C). The relationship between invasive and non-invasive CFR in the LAD artery was characterized by the following formula: CFR (TTDE-derived) = 0.52 × CFR (invasive Doppler wire-derived) + 1.09. Bland–Altman analysis showed a mean difference between invasive and non-invasive CFR of 0.03 (standard deviation ± 0.64, *p* = 0.72), with a similar pattern of agreement over a range of CFR values (Figure 2). Accordingly, a formal test showed no significant heteroscedasticity between invasive Doppler wire- and non-invasive TTDE-derived CFR (Levene’s statistic, F = 2.86, *p* = 0.10).

### 3.3. Association of Invasive and Non-Invasive CFR with Final Infarct Size

**Figure 3:**
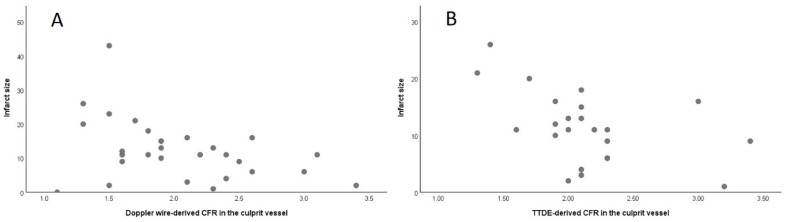
Correlation of invasive, Doppler wire-based (**A**) and non-invasive, echocardiography-derived coronary flow reserve (**B**), measured in the culprit vessel on days 3–7 after primary PCI, with final infarct size on cardiac magnetic resonance imaging.

**Figure 4:**
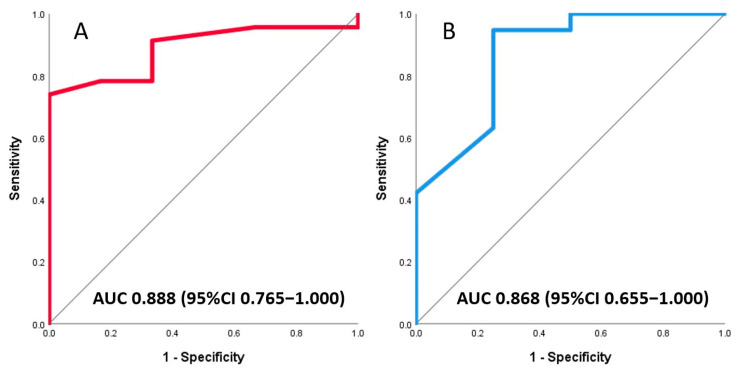
ROC analysis showing good capacity of both Doppler-wire-based (**A**) and echocardiography-derived (**B**) coronary flow reserve to predict infarct sizes ≥18% of the left ventricle.

Almost a quarter of patients (23%, 7/31) had a CMR-based infarct size ≥18% of the left ventricle. A significant correlation with infarct size was found for both invasive Doppler wire-derived CFR (r = 0.38, *p* = 0.037) and non-invasive TTDE-derived CFR (r = 0.49, *p* = 0.017), each obtained at a median of 4 days after primary PCI in the culprit vessel (Figure 3). In the receiver-operating characteristic (ROC) analysis, both invasive and non-invasive CFR were good at classifying patients according to final infarct sizes ≥18%, with an area under the curve (AUC) of 0.89 (95%CI 0.76–1.00, *p* = 0.004) for Doppler wire-derived CFR and 0.87 (95%CI 0.65–1.00, *p* = 0.023) for TTDE-derived CFR (Figure 4). A formal test did not find a significant difference between the two ROC curves. Optimal cut off values for predicting infarct sizes ≥18% were 1.85 for invasive and 1.80 for non-invasive CFR. Table 2 lists coronary physiology parameters according to the CMR-assessed infarct size.

Of note, the described degree of correlation of non-invasive and invasive CFR with infarct size was similar to what was found for hyperemic microvascular resistance (HMR) obtained on the same day as CFR (rho 0.50, *p* = 0.006). ROC curve analysis showed a comparable ability of HMR to predict final infarct sizes ≥18% (AUC = 0.74, 95%CI 0.53–0.94), with an optimal cut off of 1.9 mmHg/cm/s.

### 3.4. Association of Invasive and Non-Invasive CFR with LV Function

**Figure 5:**
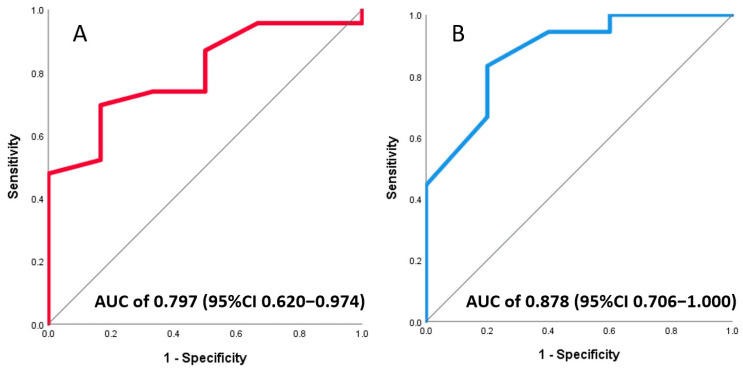
ROC analysis showed good capacity of both Doppler-wire-based (**A**) and echocardiography-derived (**B**) coronary flow reserve to predict left ventricular ejection fractions <40%.

At a median follow-up of 8 months with CMR imaging, 23% of patients (7/31) had LVEF < 40%. ROC curve analysis showed an AUC of 0.78 (95%CI 0.62–0.97) for invasive and an AUC of 0.88 (95%CI 0.71–1.00) for non-invasive CFR (Figure 5A,B). The optimal cut off to predict LVEF < 40% in the follow-up by measuring CFR on days 3–7 after primary PCI was 1.75 if assessed invasively with an intracoronary Doppler wire and 1.95 if derived from the transthoracic echocardiography Doppler. The predictive ability of CFR, measured either invasively or non-invasively, was comparable to HMR (AUC = 0.78, 95%CI 0.56–0.99) obtained on the same day.

## 4. Discussion

**Figure 6:**
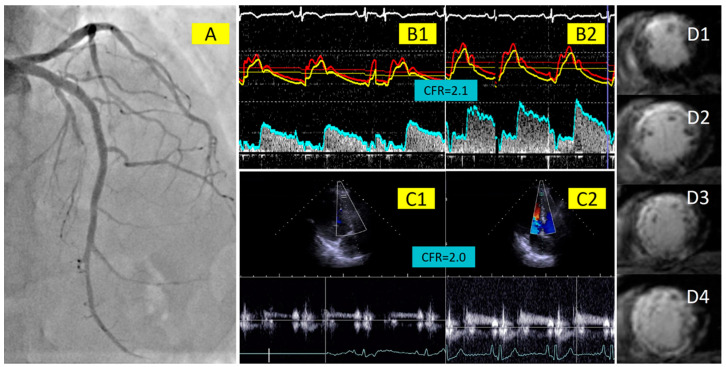
Representative images showing the recanalized LAD artery (**A**) with invasive Doppler wire-based assessment of coronary flow velocity at rest (**B1**) and in hyperemia (**B2**), and the corresponding echocardiography-derived measurements of coronary flow velocity at rest (**C1**) and during hyperemia (**C2**), which correlate with the degree of myocardial fibrosis from the mid anterior wall to the apex of the left ventricle on cardiac magnetic resonance imaging (**D1**–**D4**).

To the best of our knowledge, this is the first study to assess the correlation between non-invasive, transthoracic doppler echocardiography-derived CFR and invasive, intracoronary Doppler wire-derived CFR in patients with STEMI, several days after a successful primary PCI, and their respective ability to predict infarct size and LV function impairment (Figure 6). Our main findings are the following: First, there is a significant, albeit modest, correlation between non-invasive, TTDE-derived CFR and invasive, intracoronary Doppler wire-derived CFR. However, this significant correlation was driven by the measurements in the LAD artery, whereas there was no correlation between non-invasive and invasive CFR in the RCA artery. Second, both non-invasive and invasive CFR of the culprit artery on days 3–7 post-primary PCI had a similar ability to predict large infarct sizes and reduced LVEF, which was comparable to the predictive ability of HMR obtained on the same day.

### 4.1. Correlation between Intracoronary- and Echocardiography-Derived CFR

Prior to ours, two studies from the late 1990s showed good agreement between TTDE- and Doppler wire-derived CFR in the LAD artery in a sample of 23 and 25 patients, but they excluded patients with an acute myocardial infarction and only assessed CFR in the LAD artery [27,28]. We documented no significant correlation between CFR obtained by TTDE and an intracoronary wire in the RCA artery. Moreover, prior studies included patients with significant epicardial coronary disease and/or valvular disease. On the contrary, our study focused on patients with an MI in whom CFR was assessed after successful primary PCI and in the absence of flow-limiting lesions, as confirmed by FFR > 0.80, thus allowing for the estimation of the ability of CFR, as a measure of microvascular function, to predict infarct size and LV function. Furthermore, and unlike in our study, transthoracic echocardiography was conducted in the catheterization lab, thus allowing for a simultaneous measurement with the Doppler wire in the LAD artery [28]. This may explain a greater degree of correlation (r = 0.94) as compared with our study (r = 0.55), where TTDE was performed a few hours apart from the invasive measurement, thus offering a blinded comparison between invasive and non-invasive CFR assessment, closer to a real-life setting where coronary anatomy is often unknown to the echocardiographer.

The results of ours and the two above-described studies are important given the recently documented lack of correlation between TTDE-derived and intracoronary thermodilution-derived CFR after PCI [25]. That analysis included 174 patients with a significant epicardial LAD stenosis in the proximal segment who underwent pre- and post-PCI CFR assessment with the intracoronary thermodilution method. In addition, non-invasive, TTDE-derived CFR was measured 1 day before and 3 days after PCI [25]. The authors reported a significant but modest correlation between TTDE-derived and thermodilution-based CFR prior to PCI (r = 0.379, *p* < 0.001), when CFR measurement took into account the existing epicardial stenosis. After PCI, in the absence of significant epicardial disease, which was the clinical scenario in our study, there was no correlation between TTDE-derived and thermodilution-based CFR (r = 0.054, *p* = 0.482) [25]. This observation highlights the difference in assessing coronary flow reserve with thermodilution vs. the Doppler method. A recent study comparing the two invasive methods to assess blood flow, i.e., thermodilution- and Doppler wire-based, showed a significant correlation (r = 0.60, *p* < 0.0001), albeit with a bias towards overestimating CFR values by thermodilution compared with the Doppler method [29]. Given this documented variability in assessing coronary flow with thermodilution vs. Doppler, and the lack of correlation of the former with echocardiography-derived CFR [25], a question may be raised as to which extent non-invasive TTDE-derived CFR might be clinically utilized to assess coronary microcirculation. In confirming agreement between non-invasive, echocardiography-derived CFR and the invasive, Doppler wire-based CFR in the LAD artery, and given the prior solid evidence for predicting adverse events according to the culprit vessel intracoronary Doppler wire-based CFR [7,8,9], our study may open a window of opportunity to use the widely available transthoracic Doppler echocardiography to risk-stratify patients after STEMI.

### 4.2. Echocardiography-Derived CFR Predicts Infarct Size and LV Function after STEMI

Our results suggest that like invasive CFR, TTDE-derived CFR, measured at 3–7 days after STEMI in the recanalized culprit artery, is, to a similar extent, also able to predict final infarct size and reduced LVEF. These findings are in line with previous studies which documented the capacity of TTDE-derived parameters of coronary flow, such as diastolic deceleration time, reversal of systolic flow, and CFR to predict LV remodeling on echocardiography and myocardial perfusion defects on SPECT imaging [20,21,22,30,31]. Unlike in any of the previous studies, we measured CFR both invasively and non-invasively in the same artery and on the same day, which allowed us to compare the capacity of both methods to predict infarct size. Moreover, in contradistinction with older studies, we used cardiac magnetic resonance imaging to measure the final infarct size. This is an important point, as CMR-based infarct size has been accepted as an endpoint, often primary, in contemporary randomized trials investigating cardioprotective drugs and devices [32]. Moreover, infarct size has been associated with mortality and heart failure in a pooled patient-level analysis including 10 randomized primary PCI trials [33]. We assessed the ability of CFR to predict infarct sizes ≥18% of the LV, as it was shown to be a significant predictor of mortality and heart failure after STEMI in the same pooled analysis [33]. In addition to infarct size, both invasive and non-invasive CFR were similarly good at predicting LVEF < 40%, thus providing early stratification according to the risk of heart failure after an angiographically successful primary PCI for STEMI. This is of practical relevance, given the importance of early initiation of guide-line-directed medical therapy for heart failure [34].

### 4.3. Clinical Utility of Echocardiography-Derived CFR

Besides the direct implications for risk stratification of STEMI patients, our study may inform on the broader clinical applicability of CFR as a predictor of adverse cardiovascular events. A recent analysis pooled 79 studies which measured CFR by different methods, both invasive (thermodilution- and Doppler-derived) and non-invasive (echocardiography, PET, CMR), and reaffirmed its association with the risk of mortality across various clinical settings, including coronary artery disease (both obstructive and non-obstructive), heart failure, valvular heart disease, and diabetes [3]. More specifically, a recent large iPOWER study investigated the potential of TTDE-derived CFR in the LAD artery to predict adverse cardiovascular events in a population of 1853 women (CFR was successfully obtained in 91% of the cases) with angina but without obstructive coronary disease [35]. CFR was associated with all-cause death, and the risk of myocardial infarction and heart failure during the 4.5 years of follow-up. Interestingly, the optimal CFR cut off value to discriminate between patients with and without adverse events was found to be 2.25 in the iPOWER study [35]. This is important in the light of simplifying clinical adoption by providing a relevant cut off value. In the field of invasive coronary physiology, discussions are ongoing to implement a new wire-based CFR threshold of 2.5, a change from the currently recommended value of 2.0 [29]. According to the relationship defined in our study [(TTDE-derived) = 0.52 × CFR (invasive Doppler wire-derived) + 1.09], for a Doppler-wire based CFR value of 2.5, a TTDE measurement would produce a CFR value of 2.4, which is also close to the cut off defined in the iPOWER study.

In summary, the results of our study seem to further support the general concept of multimodality imaging for cardiovascular risk assessment [36]. We showed that transthoracic Doppler echocardiography may be used to assess CFR in the recanalized IRA several days after successful primary PCI, thus reflecting the functional state of coronary microcirculation. The so-obtained CFR values correlate with infarct size on CMR, which, on its end, is predictive of heart failure and mortality [33]. Therefore, using different imaging modalities, such as TTDE and CMR, in patients with STEMI may improve our ability to stratify patients according to the risk of death and heart failure, despite successful primary PCI.

However, future research is needed to establish a direct relationship between TTDE-derived CFR in the recanalized IRA and clinical outcomes after STEMI. This is needed in order to fully utilize multimodality imaging as a stratification tool in patients with STEMI. Namely, unlike the detection of final infarct size on CMR, the early stratification by TTDE-derived CFR may help to guide treatment strategies early after reperfusion, which may ultimately decrease infarct size and improve prognosis.

### 4.4. Limitations

There are several limitations, most of which are inherent to a single-center, observational study design. First, we included a relatively small number of patients, which is nevertheless comparable to previous similar studies that evaluated the agreement among invasive indices of coronary microvascular dysfunction, with the hitherto largest study being a combined analysis from four centers that included 149 patients. Second, although patients did abstain from caffeine for at least 12 h prior to the measurements, there are still several sources of variability in both the invasive and non-invasive CFR assessment, including the discrepancy between intracoronary wire position and the ultrasound probe, especially in the cases of diffuse distal disease and/or a large diagonal branch. However, we thought that our design, where echocardiography assessors were unaware of detailed coronary anatomy, would better replicate clinical practice when compared to some of the previous studies which compared CFR values obtained by simultaneous echocardiography and invasive, wire-based measurements in the catheterization laboratory. Third, due to infrastructural constraints, especially during the COVID-19 pandemic, seven patients who underwent invasive coronary physiology assessment did not undergo planned TTDE CFR measurement. Fourth, Doppler signals by transthoracic echocardiography could not be obtained in three vessels (two RCA and one LAD) in three different patients. Fifth, the CMR-based infarct size of 18% was selected as clinically meaningful according to the hitherto largest pooled analysis that examined the impact of infarct size on clinical outcomes [33]. However, in that study, CMR was performed within 1 month of primary PCI, whereas in our study, CMR was performed at a median of 8 months. As infarct size as a % of the LV may decrease over the early period after STEMI, we opted for maintaining this cut off as a conservative estimate of poor prognosis.

## 5. Conclusions

Transthoracic Doppler echocardiography-derived CFR correlates with the Doppler wire-based CFR in the LAD artery, but not in the RCA artery, in patients with STEMI, on days 3–7 after successful primary PCI. Both invasive CFR and its non-invasive correlate, TTDE-derived CFR, are significantly and to a similar extent associated with final infarct size and reduced LVEF on CMR, and can be used to risk-stratify patients after STEMI treated with angiographically successful primary PCI.

## Figures and Tables

**Table 1:** Clinical characteristics of the overall population.

	Age, Years	59 (53–62)
Clinical presentation and demographics	Male, %	86
	Time to reperfusion, h	180 (120–252)
	Systolic blood pressure, mmHg	125 ±15
	Diastolic blood pressure, mmHg	80 ± 11
	Heart rate, beats/min	75 ± 14
	Hypertension, %	62
	Dyslipidemia, %	80
	Diabetes, %	17
	Smoking, %	65
	BMI, kg/m^2^	28 ± 4
	Maximum CK, U/L	2718 ± 1848
	Maximum high-sensitivity Troponin T, ng/L	6119 ± 3450
Procedural characteristics	Infarct-related artery, %	69 (LAD)
		31 (RCA)
	Transradial access, %	100
	Manual thrombectomy, %	17
	Glycoprotein inhibitor use, %	13
	Direct stenting, %	10
	Total stented length, mm	26 (21–47)
	Post-dilatation, %	58
Echocardiography during index hospitalization	Left ventricular ejection fraction, %	45 ± 11
	End-diastolic diameter, mm	54 ± 5
	End-systolic diameter, mm	38 ± 5
	Incomplete (≤70%) ST-segment resolution on ECG	48

**Table 2:** Coronary physiology and cardiac magnetic resonance parameters according to the infarct size.

		CMR-Based Infarct Size <18% (*n* = 23)	CMR-Based Infarct Size ≥18% (*n* = 6)	*p*-Value
Invasive Doppler wire-based coronary physiology	APV resting, cm/s	21.17 ± 6.97	22.17 ± 8.08	0.77
	APV hyperemia, cm/s	43.78 ± 11.10	32.17 ± 8.63	0.02
	CFR	2.16 ± 0.55	1.52 ± 0.20	0.01
	Pa resting, mmHg	93.13 ± 10.84	85.50 ± 5.54	0.11
	Pd resting, mmHg	89.78 ± 12.20	77.83 ± 6.97	0.03
	Pa hyperemia, mmHg	94.43 ± 16.96	84.00 ± 7.77	0.16
	Pd hyperemia, mmHg	87.52 ± 18.63	76.83 ± 12.71	0.20
	FFR	0.93 ± 0.05	0.89 ± 0.04	0.11
	HMR	2.16 ± 0.68	2.60 ± 0.46	0.12
Non-invasive transthoracic Doppler echocardiography-based coronary physiology *	APV resting, m/s	0.26 ± 0.04	0.30 ± 0.13	0.54
	APV hyperemia, m/s	0.59 ± 0.14	0.49 ± 0.18	0.37
	CFR	2.25 ± 0.46	1.62 ± 0.36	0.02
Cardiac magnetic resonance ^†^	LVEDV, mL **	144.00 ± 58.00	222.71 ± 98.00	<0.01
	LVEDV indexed, mL/m^2^ **	76 ± 35	96 ± 52	<0.01
	LVESV, mL **	77 ± 43	131 ± 77	0.01
	LVESV indexed, mL/^2^ **	33 ± 20	65 ± 40	<0.01
	LVEF, %	54 ± 7	36 ± 8	<0.01
	Infarct size, % of the LV **	11.00 ± 9.00	21.00 ± 7.00	<0.01

* 19 patients with infarct size < 18%, and 4 in the group of patients with infarct size ≥18%. ^†^ 24 patients with infarct size < 18%, and 7 in the group of patients with infarct size ≥18%. ** presented as median (interquartile range).

## Data Availability

The original contributions presented in the study are included in the article, further inquiries can be directed to the corresponding author.
